# Efficacy and safety of ginkgo biloba extract combined with donepezil hydrochloride in the treatment of Chinese patients with vascular dementia: A systematic review meta-analysis

**DOI:** 10.3389/fphar.2024.1374482

**Published:** 2024-07-03

**Authors:** Liangyi Xiao, Jie Tang, Huizhong Tan, Yao Xie, Shiliang Wang, Le Xie, Dahua Wu

**Affiliations:** ^1^ Graduate School, Hunan University of Chinese Medicine, Changsha, China; ^2^ Department of Acupuncture Rehabilitation, Changsha Hospital of Traditional Chinese Medicine, Changsha, China; ^3^ Department of Neurology, Hunan Provincial Hospital of Integrated Traditional Chinese and Western Medicine (Hunan Academy of Chinese Medicine Affiliated Hospital), Changsha, China

**Keywords:** ginkgo biloba extract, donepezil, vascular dementia, meta-analysis, systematic review

## Abstract

**Objective:** To conduct a meta-analysis of the effectiveness and safety of ginkgo biloba extract combined with donepezil hydrochloride vs. donepezil for the treatment of vascular dementia (VaD).

**Methods:** Four English databases (PubMed, EMBASE, Web of Science, Cochrane Library) and four Chinese databases [the China National Knowledge Infrastructure Wanfang DATA, the Chongqing VIP Database (VIP), China Biomedical Database (CBM)] were manually searched for literature published from dates of the inception of the databases to September 2023. The randomized controlled trials (RCTs) of ginkgo biloba extract with donepezil hydrochloride vs. donepezil for the treatment of VaD were included. Relevant literature was screened, and the data in the included studies were extracted for quality assessment according to the Risk of bias tool.

**Results:** A total of 1,309 participants were enrolled in the 15 RCTs. Of these, 656 participants were in the experimental group (ginkgo biloba extract combined with donepezil) and 653 participants were in the control group (donepezil).The results showed that combination therapy was superior to donepezil alone, and there were statistically significant differences in several outcomes including RR in change for total effective rate (1.28, 95% confidence intervals 1.20, 1.38, *p* < 0.001), MD in change for Mini-Mental State Examination score (2.98, 95%CI 2.31, 3.65, *p* < 0.001), Barthel Index score (8.55,95%CI 1.11, 15.99, *p* = 0.024), Activity of Daily Living Scale (ADL)score (10.11,95% CI 7.16,13.07,*p* < 0.001).

**Conclusion:** Ginkgo biloba extract combined with donepezil dramatically improved the total effective rate, MMSE, BI and ADL scores, and decreased homocysteine (HCY), plasma viscosity (PV), whole blood viscosity at high cut (BVH) and whole blood viscosity at low cut (BVL) in VaD patients, while the effect on mean flow velocity and pulse index (PI) of middle cerebral artery (MCA) is not obvious. However, more relevant high-quality RCTs are needed to validate these results.

**Systematic Review Registration:** Identifier CRD42023474678.

## 1 Introduction

Vascular dementia (VaD), which caused by cerebrovascular disease, is a common type of dementia in clinical practice. It becomes the second most common type of dementia after Alzheimer’s disease (AD) in recent years, accounting for about 15% of dementia cases (Kalaria, 2018; OBrien and Thomas, 2015). The main clinical manifestations include cognitive, psychological, and behavioral symptoms, such as repetitive questioning, restlessness, depression, apathy, confusion, aggression, sleep disorders, and various misbehaviors (Kales et al., 2015). Consequently, the occurrence of VaD has a negative impact on the living quality and safety of patients, and brings a huge burden to the family and society, such as self-care problems or communication disorders.

Currently, there is no uniformed standard on the treatment of dementia. Generally recognized therapeutic drugs include acetylcholinesterase inhibitors and NMDA receptor antagonists (Farooq et al., 2017). Donepezil, galantamine, memantine, etc., are commonly used in clinical treatment of the disease, which are proved to improve the cognitive function on patients with different stages (Orgogozo et al., 2002; Wilkinson et al., 2003; Auchus et al., 2007).

In addition, since ginkgo biloba extract has been reported as a natural medicine in the 1980s (Subhan and Hindmarch, 1984), it is widely used in the management of cardiovascular disease and coronary heart disease (Shaito et al., 2020). Morever, experts have reached a consensus on the use of ginkgo biloba extract to treat cognitive disorders (Kandiah et al., 2019). Flavonoids and terpenoids are considered as pharmacologically active components of Ginkgo biloba (Smith et al., 1996; Shi et al., 2009). At present, Ginkgo biloba preparation mainly includes oral preparation and injection preparation. The oral formulation contains 19.2 mg of total flavonoid glycoside and 4.8 mg of terpenoid lactone or 9.6 mg of total flavonoid glycoside and 2.4 mg of terpenoid lactone. Shuxuening is a commonly used ginkgo biloba injection. Every 5 mL Shuxuening contains 17.5 mg of ginkgo biloba extract (4.2 mg of total flavonoid glycosides and 0.70 mg of ginkgolide) (Yang et al., 2016). The neuroprotective mechanism of ginkgo biloba extract is clear in two aspects: on the one hand, it can reduce the damage caused by platelet activating factor (PAF) during cerebral ischemia by antagonizing PAF; On the other hand, it can remove free radicals in the body, produce antioxidant effect, and reduce the damage of brain tissue caused by excessive free radicals produced in the body during cerebral ischemia (Noorbala and Akhondzadeh, 2006). Besides, some studies have mentioned that ginkgo biloba extract can treat dementia patients by regulating serum HCY concentration and hemorheology (Diamond et al., 2000).

A lot of clinical studies have proven its effectiveness and safety in the treatment of dementia, both AD and VaD (Ihl et al., 2012). Besides, a meta-analysis indicated that ginkgo biloba extract could improve cognitive function and the ability to live daily in people with VaD, and this meta-analysis included RCTs of ginkgo biloba extract combined with VaD drugs and control group with VaD drugs alone. (The drugs for VaD treatment mainly include donepezil, nimodipine, huperzine A, oxiracetam, piracetam, butylphthalide and other drugs that promote microcirculation and improve cognition. Ginkgo biloba extract includes common ginkgo biloba products, namely, tablets, capsules, and soft capsules (Zhan et al., 2021). However, there is still a lack of meta-analysis on conventional western medicine, represented by donepezil, compared with ginkgo biloba extract combined with donepezil for VaD treatment. Therefore, to synthesize more evidence for the role of ginkgo biloba combined with donepezil in the treatment of this disease, we included articles published in recent years in a more comprehensive meta-analysis evaluating the efficacy and safety of this regimen.

## 2 Methods

The protocol of this review has been registered in the International Prospective Register Of Systematic Reviews, and the approval number for registration is CRD42023474678. It was reported following the statement guidelines of preferred reporting items for systematic reviews and meta-analyses protocols (Moher et al., 2015).

### 2.1 Search strategy

The literature search was conducted in database, including four English databases (PubMed, EMBASE, Web of Science, Cochrane Library) and four Chinese databases [the China National Knowledge Infrastructure (CNKI), Wanfang DATA, the Chongqing VIP Database (VIP), China Biomedical Database (CBM)], were manually searched for literature published from dates of the inception of the databases to 7 September 2023(Beijing time).In order to comprehensively search and obtain relevant literature, a manual search was carried out using the following search terms and their variants: ginkgo biloba, ginkgo leaf extract, donepezil and VaD. Detailed retrieval strategies are provided in Supplementary Material S1.

### 2.2 Eligibility criteria

#### 2.2.1 Inclusion criteria

(1) Participants: Patients with a clinical diagnosis of VaD were considered regardless of nationality, race, gender, occupation, or educational background.
Although the causes of VaD are not limited, all patients should be diagnosed with VaD according to at least one of the current or past VaD definitions or guidelines, such as:
① Diagnostic and Statistical Manual of Mental Disorder fourth, DSM-IV (Gmitrowicz and Kucharska, 1994).
② Draft diagnostic criteria for vascular dementia, Chinese Medical Association Branch of Neurology (Branch CMAN, 2002).
③ Rockwood clinical diagnostic criteria for VCI (Erkinjuntti and Rockwood, 2003)
④ Discussion on clinical diagnostic Criteria of Vascular Dementia (Jia and Wang, 2010).
(2) Intervention: The experimental group was given ginkgo biloba preparation combined with donepezil.
(3) Comparision: The control group was only given donepezil.
(4) Outcomes: One of the following outcomes was reported: Primary outcome including total effective rate and Mini-Mental State Examination (MMSE) score, Barthel Index (BI) score and Activity of Daily Living Scale (ADL) score; secondary outcome including homocysteine (HCY), hemorheological indicators [whole blood viscosity at high cut (BVH), whole blood viscosity at low cut (BVL), plasma viscosity (PV), fibrinogen (FIB)], mean flow velocity and pulse index (PI) of middle cerebral artery (MCA) and adverse events as safety outcomes.
(5) Study design:Randomized controlled trials (RCTs) involving patients diagnosed with VaD.

#### 2.2.2 Exclusion criteria

(1) Reviews, case reports, research protocols or conference papers.
(2) Animal and *in vitro* studies.
(3) Duplication and lack of access to full texts.
(4) Literature with outcome indicators or data errors could not be extracted.

Two reviewers Liangyi Xiao and Jie Tang independently screened the literature according to the above criteria, and the different opinions encountered during the research screening process were resolved through discussion or by the third reviewer Yao Xie.

### 2.3 Data extraction and quality assessment

Two reviewers (Liangyi Xiao and Jie Tang) independently extracted data from the final included literature, including first author, publication year, country, intervention and control measures, duration of treatment, basic information about study subjects, outcome measures, etc. Included studies will be evaluated by two independent reviewers (Liangyi Xiao and Jie Tang) using the Cochrane Bias Risk Assessment Tool (RoB2.0) (Sterne et al., 2019) on five aspects of included randomised controlled studies: Bias arising from randomization, bias from deviations from established interventions, bias from missing outcome data, bias from outcome measurement, bias from selective reporting of results. For each study, an independent quality assessment was conducted by two researchers, who rated the five aspects as “low risk,” “high risk,” and “possibly risky.” The diverging literature was evaluated through discussion or the suggestion of a third investigator, and the evaluation results were shown in the bias risk map.

### 2.4 Data integration and statistical analysis

The main outcome indicator are total effective rate, MMSE score, BI score and ADL score. The secondary outcome indicator are HCY, hemorheological indicators (BVH, BVL, PV, FIB), mean flow velocity and PI of MCA and adverse events as safety outcomes. We performed a meta-analysis using stata15.1. For continuous data, when using the same scale, weighted mean differences (WMD) were calculated and 95% confidence intervals (CI) were reported. For binary categorical variables, RR was used as the effect index for meta-analysis. Heterogeneity tests were based on *p*-value obtained from Q tests combined with the I^2^ statistic. Among them, the I^2^ statistic is an important indicator of heterogeneity, the value of 25%, 50% and 75%, representing low, medium and high heterogeneity (Higgins et al., 2003). If there is no significant heterogeneity between each study, i.e., I^2^ < 50% and *p* > 0.1, a meta-analysis is performed using a fixed-effect model (Mantel-Haenszel method). Instead, a random effects model (DerSimonian-Laird method) will be used.Subgroup analysis and regression analysis were based on efficacy evaluation criteria and treatment duration to determine the magnitude and source of heterogeneity among studies. Sensitivity analysis was used to evaluate the robustness of the meta-analysis results. Funnel plots were created to assess whether publication bias existed in the included literature, and Egger or Begg methods were used for statistical testing (the number of studies should be ≥ 5). For the results with significant publication bias, the shear compensation method was used to measure the impact of publication bias on the results.

## 3 Results

### 3.1 Literature screening results and flow charts

**FIGURE 1:**
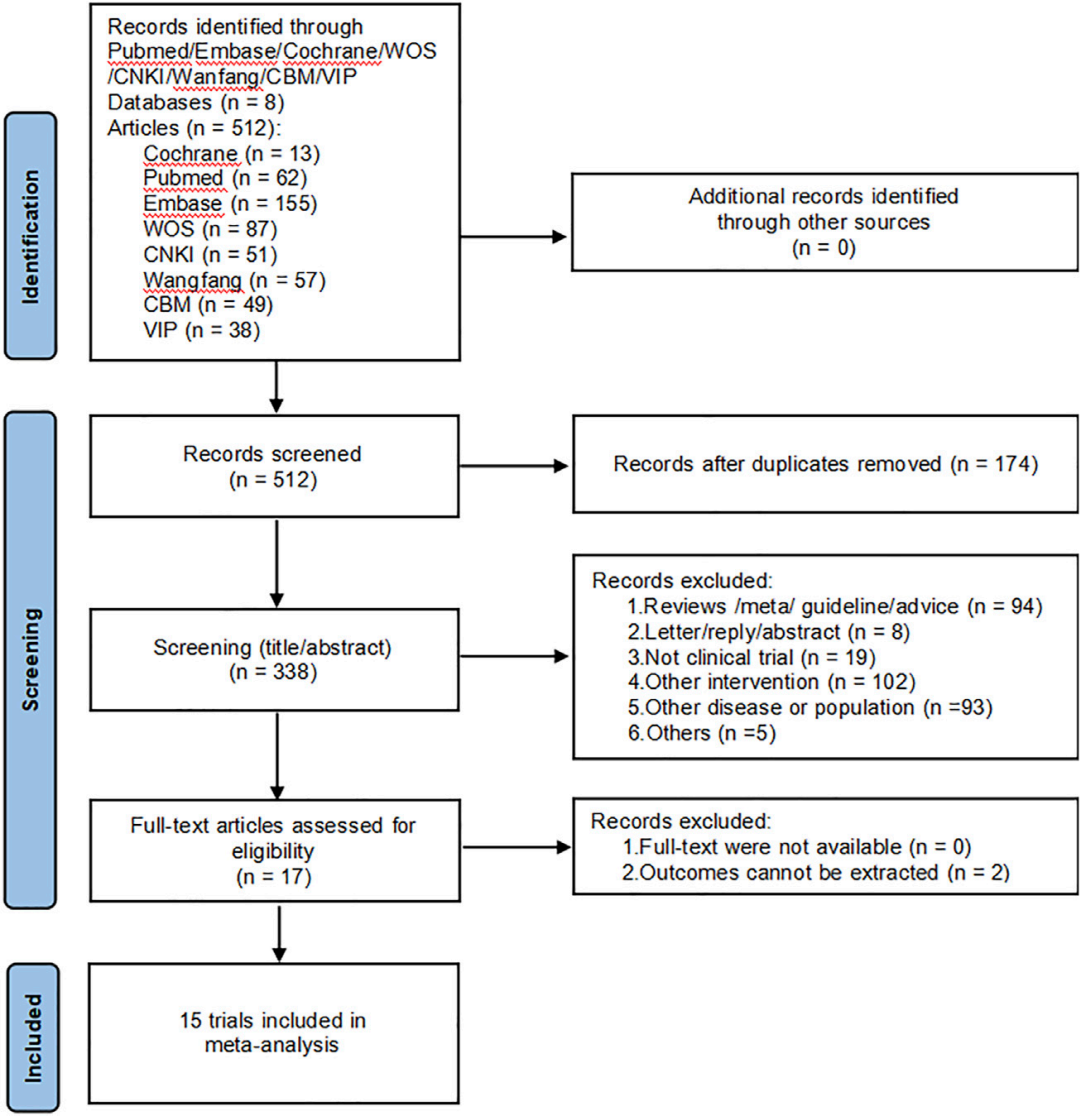
The PRISMA flowchart of the literature search and selection.

A total of 512 papers were retrieved from the initial database search, and no additional studies were identified from the reference scan. After removing duplicates, 338 articles were examined by title and abstract. Of these, 323 articles were excluded because they did not meet the inclusion criteria, 17 articles have been carefully reviewed for full text. Finally, 15 studies were included in this meta-analysis (Guo, 2010; Chen, 2011; Bao and He, 2012; Yu, 2014; He et al., 2016; Guo, 2018; Guan, 2019; Xu, 2019; Chen and Li, 2020; Gao et al., 2020; Miao, 2020; Zheng, 2020; Duan et al., 2021; Tong, 2021; Shen et al., 2023). Figure 1 shows the literature screening process.

### 3.2 Studies’ characteristics

The basic characteristics of the included studies are in Table 1.

**TABLE 1:** Basic Characteristics of the included studies.

References	Sample size		Country	Male/female		Age(y)		Intervening measure		Main outcome	Treatment course
	E	C		E	C	E	C	E	C		
He et al. (2016)	50	50	China	32/18	30/20	67.8 ± 3.0	67.1 ± 2.9	GBE*^1^: 2 pills, orally, 3 times daily; DH: 1 pill, once daily	DH: 1 pill, once daily	①②	12 weeks
Gao et al. (2020)	60	60	China	46/14	48/12	64.2 ± 10.3	65.8 ± 10.2	GBE*^1^: 1 pills, orally, 3 times daily; DH: 1 pill, once daily	DH: 1 pill, once daily	①②③⑫	12 weeks
Chen and Li. (2020)	31	29	China	17/14	17/12	68.68 ± 5.17	68.17 ± 5.87	GBE*^2^: 10mL, orally, 3 times daily; DH: 2 pills, once daily	DH: 2 pills, once daily	②③⑫	12 weeks
Guo (2018)	29	29	China	16/13	15/14	75.33 ± 7.1	75.14 ± 7.66	GBE**: 20mL, iv drip, twice daily; DH: 1 pill, once daily	DH: 1 pill, once daily	①②③⑫	4 weeks
Duan et al. (2021)	40	40	China	24/16	23/17	64.0 ± 6.0	65.0 ± 6.0	GBE**: 10mL, iv drip, daily; DH: 1 pill, once daily	DH: 1 pill, once daily	①②④⑫	8 weeks
Zheng (2020)	50	50	China	27/23	26/24	63.04 ± 6.83	63.13 ± 7.8	GBE*^3^: 4 pills, orally, 3 times daily; DH: 1 pill, once daily	DH: 1 pill, once daily	①②④	12 weeks
Tong (2021)	34	34	China	18/16	19/15	65.39 ± 7.82	64.8 ± 7.91	GBE*^1^: 2 pills, 3 times daily; DH: 1 pill, once daily	DH: 1 pill, once daily	③	4 weeks
Shen et al. (2023)	47	47	China	24/23	25/22	75.48 ± 4.02	75.34 ± 3.96	GBE*^1^: 1 pill, orally, 3 times daily; DH: 1 pill, once daily	DH: 1 pill, once daily	⑤⑥⑦⑧⑫	12 weeks
Xu (2019)	43	43	China	28/15	27/16	63.86 ± 4.71	64.11 ± 4.52	GBE*^1^: 2 pills, orally,3 times daily; DH: 1 pill, once daily	DH: 1 pill, once daily	①②③⑥⑦⑨⑫	12 weeks
Yu (2014)	35	35	China	17/18	15/20	63.1 ± 7.7	62.5 ± 7.3	GBE**: 20mL, iv drip, daily; DH: 2 pill, once daily	DH: 1 pill, once daily	①②④⑩⑪	12 weeks
Guan (2019)	46	46	China	29/17	31/15	63.59 ± 4.04	64.05 ± 3.81	GBE**: 20mL, iv drip, daily; DH: 1 pill, once daily	DH: 1 pill, once daily	②④⑫	12 weeks
Miao (2020)	79	79	China	42/37	39/40	62.33 ± 4.45	66.14 ± 2.65	GBE*^1^: 1 pill, orally, 3 times daily; DH: 1 pill, once daily	DH: 1 pill, once daily	①②⑤⑫	8 weeks
Guo (2010)	30	30	China	NM	NM	NM	NM	GBE**: 20mL, iv drip, daily; DH: 1 pill, once daily	DH: 1 pill, once daily	②⑥⑦⑧⑨⑩⑪	4 weeks
Bao and He. (2012)	40	40	China	28/13	25/16	67 ± 10	69 ± 11	GBE*^1^: 2 pills, orally, 3 times daily; DH: 1 capsule, once daily	DH: 1 capsule, once daily	①②③	12weeks
Chen (2011)	41	40	China	24/17	23/L7	63.1 ± 8.1	62.4 ± 7.8	GBE**: 20mL, iv drip, daily; DH: 1 pill, once daily	DH: 1 pill, once daily	②	12 weeks

①Total effective rate ②MMSE, score ③BI, score ④ADL, score ⑤HCY ⑥BVH ⑦BVL ⑧PV ⑨FIB ⑩mean flow velocity of MCA ⑪PI, of MCA; ⑫adverse events; NM: not mentioned; GBE: ginkgo biloba extract; DH: donepezil hydrochloride; E: experimental group; C:control group; *^1^: tablet; *^2^: oral liquid; *^3^: dropping pill; **: intravenous drip.

The fifteen included studies involved 1,309 patients from China, ranging in age from 62.33 ± 4.45 to 75.48 ± 4.02 years old. Of these, 656 participants were in the experimental group (ginkgo biloba extract combined with donepezil) and 653 participants were in the control group (donepezil), published from 2010 to 2023. The size of samples included in the study varies from 58 cases to 158 cases. The outcome indicators reported are as follows: total effective rate (n = 9), MMSE score (n = 13), BI score (n = 6), ADL score (n = 4), HCY (n = 2), BVH(n = 3), BVL (n = 3), PV(n = 2), FIB(n = 2) and mean flow velocity and PI (n = 2) of MCA (n = 2). In addition, the treatment course ranges from 4 weeks to 12 weeks.

### 3.3 Quality evaluation

**FIGURE 2:**
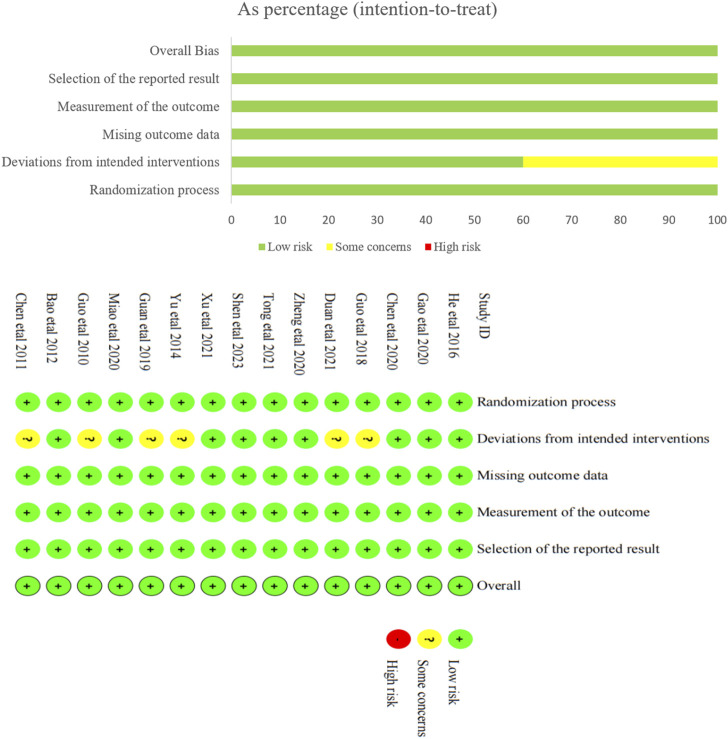
Assessment of risk of bias in the included studies (RCTs).

The results of bias risk assessment for the 15 included studies are shown in Figure 2. Among the bias generated during randomization, all the included studies were randomly assigned, which was a low risk bias. Of the bias from the established interventions, six studies were assessed as potentially risky because they did not implement double-blind method, but were assessed as low-risk bias after reasonable analytical methods provided, while the remaining nine studies were assessed as low-risk. All studies had low risk bias in missing outcome data and measurement outcomes. It was not clear from all studies whether there was selective reporting, and this risk of bias is a possible risk. Taken together, the risk of bias in the included literature was small.

### 3.4 Results of meta-analysis

#### 3.4.1 Total effective rate

**FIGURE 3:**
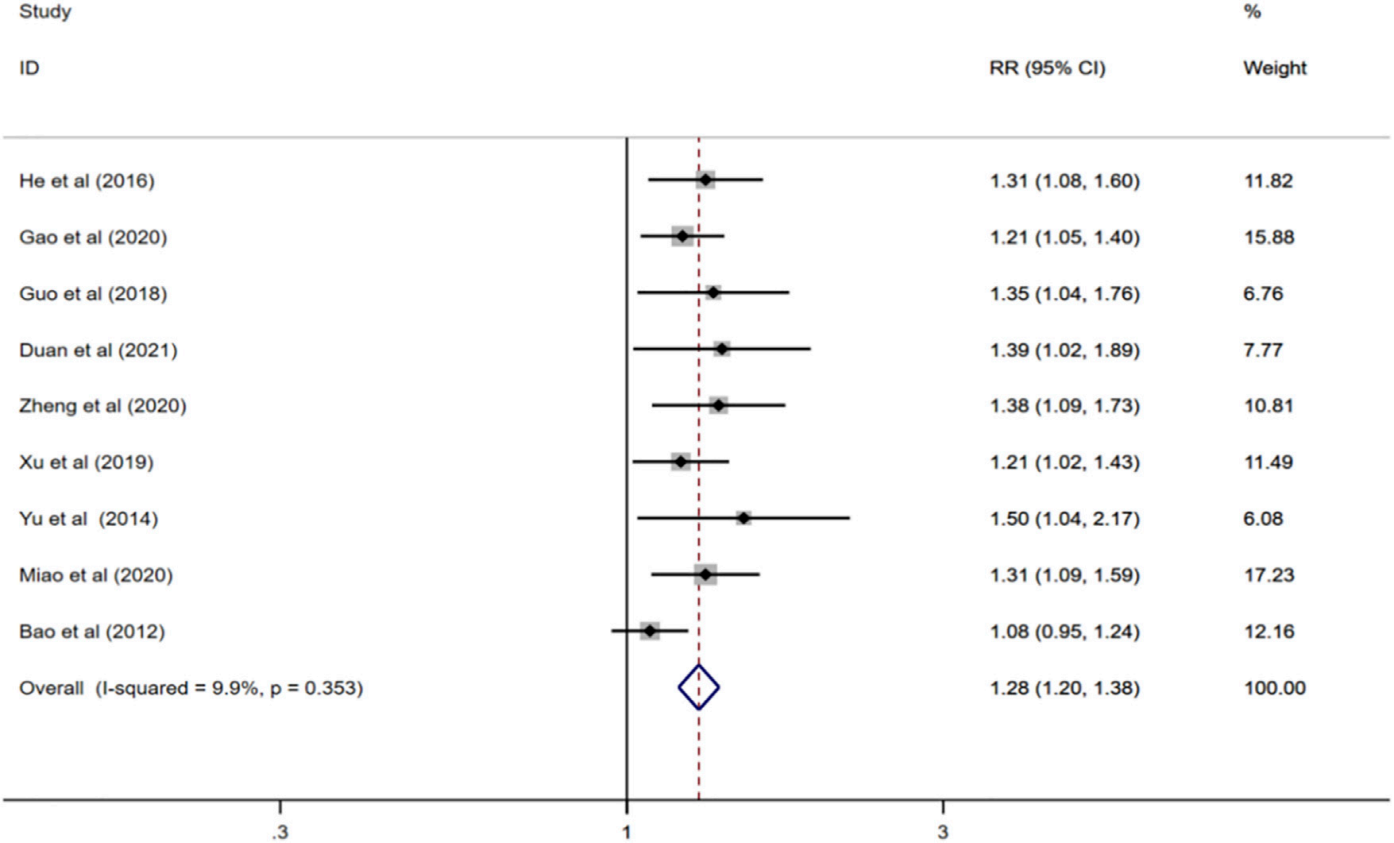
Forest plot of total effective rate.

Nine reported total effective rate (Bao and He, 2012; Yu, 2014; He et al., 2016; Guo, 2018; Xu, 2019; Gao et al., 2020; Miao, 2020; Zheng, 2020; Duan et al., 2021), and meta-analysis was performed using a fixed-effect model (I^2^ = 9.9%, *p* = 0.353). The results showed that compared with donepezil, ginkgo biloba extract combined with donepezil could improve the total effective rate (RR = 1.28; CI:1.20 to1.38; *p* < 0.001) Figure 3.

#### 3.4.2 MMSE score

**FIGURE 4:**
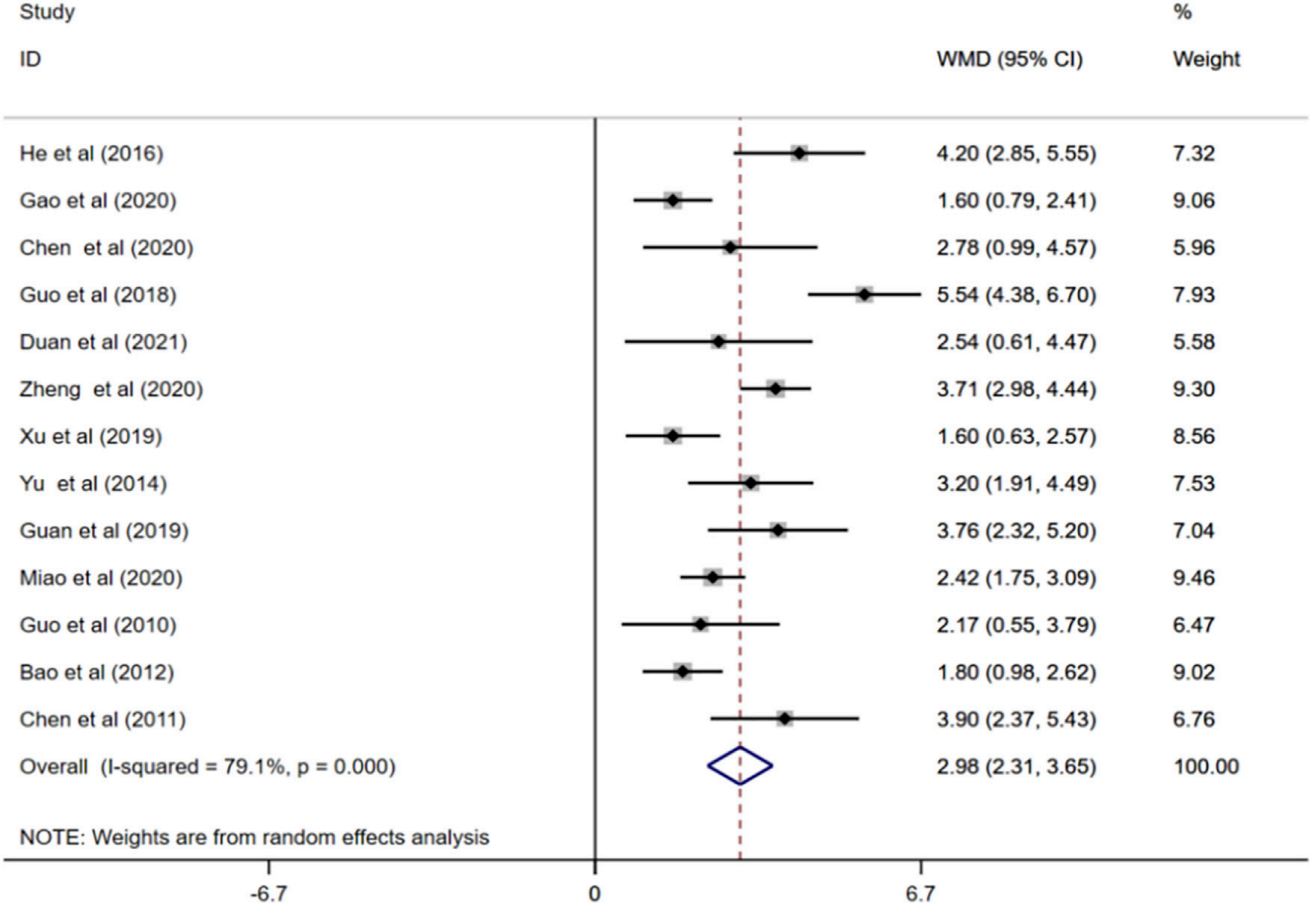
Forest plot of MMSE score.

MMSE scores were reported in 13 studies (Guo, 2010; Chen, 2011; Bao and He, 2012; Yu, 2014; He et al., 2016; Guo, 2018; Guan, 2019; Xu, 2019; Chen and Li, 2020; Gao et al., 2020; Miao, 2020; Zheng, 2020; Duan et al., 2021). Random effects model showed that treatment with ginkgo biloba extract combined with donepezil remarkably increased patients’ MMSE scores compared to treatment of donepezil alone. (WMD = 2.98; 95%CI:2.31 to 3.65; *p* < 0.001; I^2^ = 79.1%, *p* < 0.001), Figure 4.

#### 3.4.3 BI score

**FIGURE 5:**
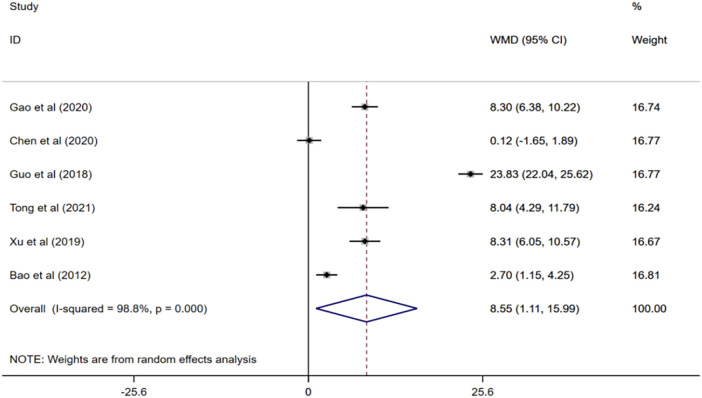
Forest plot of BI score.

Six randomized controlled trials (Bao and He, 2012; Guo, 2018; Xu, 2019; Chen and Li, 2020; Gao et al., 2020; Tong, 2021) evaluated the effects of ginkgo biloba extract combined with donepezil on BI scores in patients with VaD. Meta-analysis of the random effects model showed that BI score of the experimental group was higher than that of the control group, and the difference was statistically significant (WMD = 8.55; 95% CI: 1.11 to 15.99; I^2^ = 98.8%, *p* < 0.001) Figure 5.

#### 3.4.4 ADL score

**FIGURE 6:**
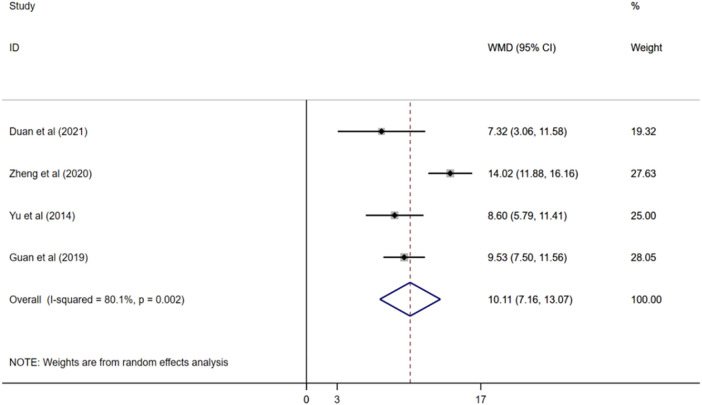
Forest plot of ADL score.

Four studies reported ADL scores (Yu, 2014; Guan, 2019; Zheng, 2020; Duan et al., 2021), and a random effects model was used for meta-analysis (I^2^ = 80.1%, *p* < 0.01). The results demonstated that compared with donepezil, ginkgo biloba extract combined with donepezil could improve ADL score observably. (WMD = 10.11; 95%CI:7.16 to13.07; *p* < 0.001) Figure 6.

#### 3.4.5 Homocysteine

**FIGURE 7:**
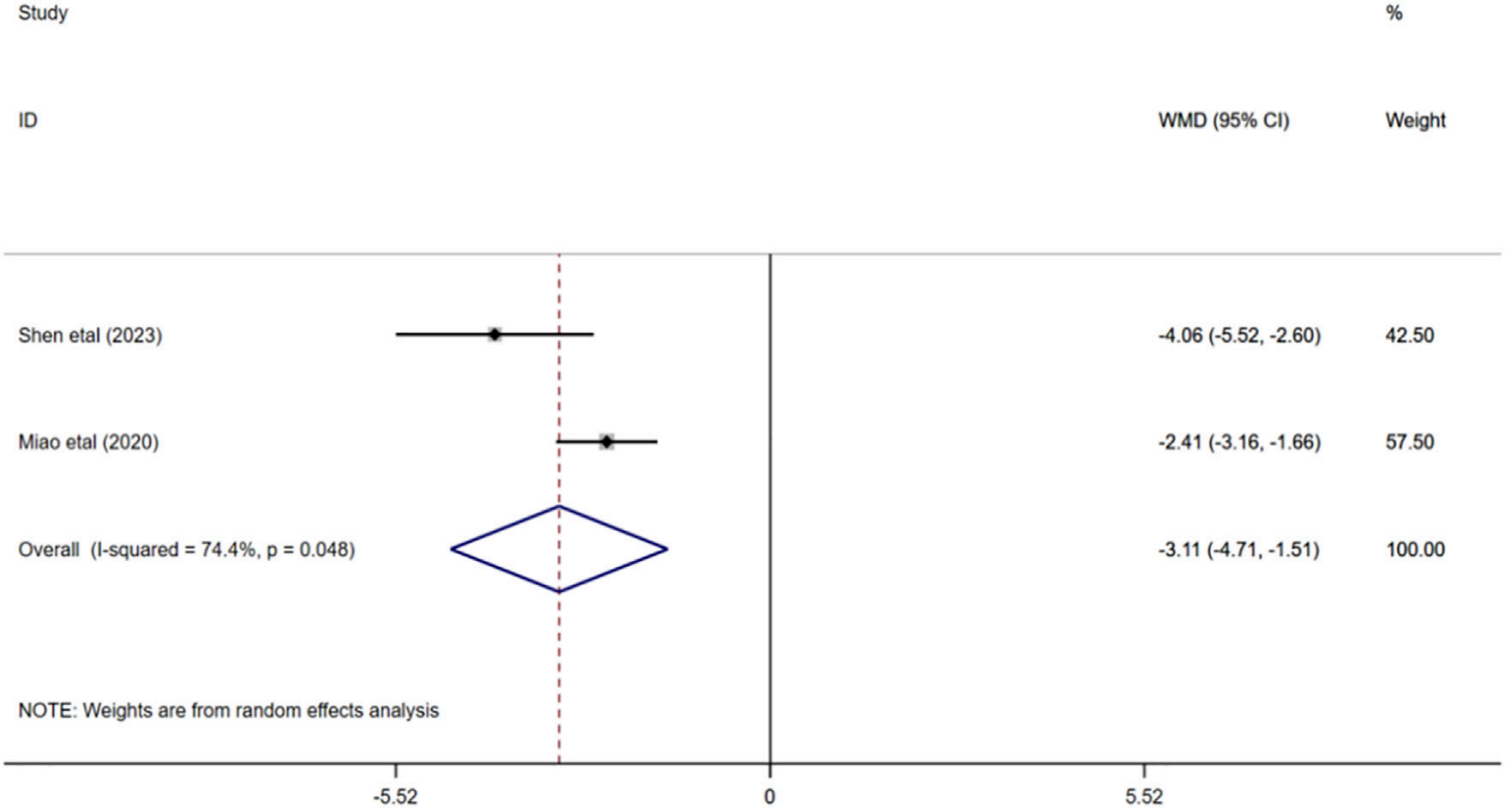
Forest plot of HCY.

A total of 252 participants were enrolled from two studies (Miao, 2020; Shen et al., 2023), with 126 participants in the experimental group and 126 in the control group. Q test for heterogeneity showed heterogeneity in effect size (I^2^ = 74.4%, *p* < 0.05), indicating high heterogeneity between studies. In consequence, the random effects model is used for analysis. The results of meta-analysis manifested that HCY in experimental group was significantly lower than that in control group (WMD = −3.11; 95% CI: -4.71 to −1.51; *p* < 0.001) Figure 7.

#### 3.4.6 Hemorheological indicators

Three randomized controlled trials (Guo, 2018; Xu, 2019; Shen et al., 2023) evaluated the effects of ginkgo biloba extract combined with donepezil on hemorheology in patients with VaD. In terms of the influence on BVH and BVL, the result demonstated that compared with donepezil, ginkgo biloba extract combined with donepezil could reduce the BVH and BVL (WMD = −1.12; 95% CI: −1.88 to −0.36; *p* < 0.01, WMD = −2.15; 95% CI: −2.48 to −1.83; *p* < 0.001, respectively).

**FIGURE 8:**
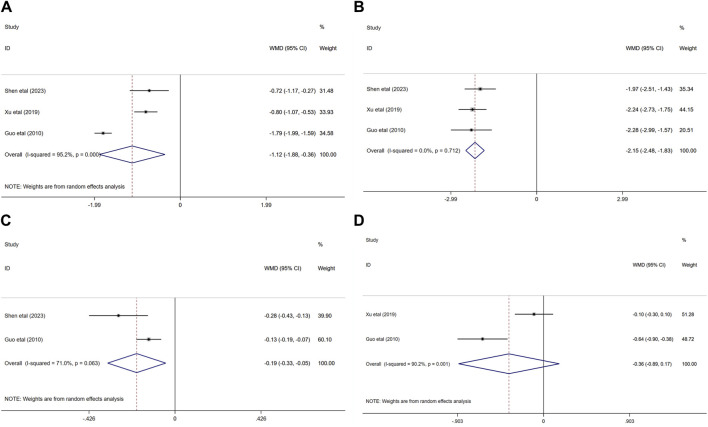
Forest plot of **(A)** BVH, **(B)** BVL, **(C)** PV, **(D)** FIB.

Two of the three studies researched the effects of combination medication on FIB(33, 37) and PV(32, 37) in patients with VaD. The results indicated that the PV of the experimental group was significantly lower than that of the control group (WMD = −0.19; 95% CI: −0.33 to −0.05; *p* < 0.05). Nevertheless, FIB decreased in both experimental group and control group (WMD = −0.36; 95% CI: −0.89 to −0.17; *p* > 0.05) Figure 8.

#### 3.4.7 Mean blood flow velocity and pulse index of MCA

**FIGURE 9:**
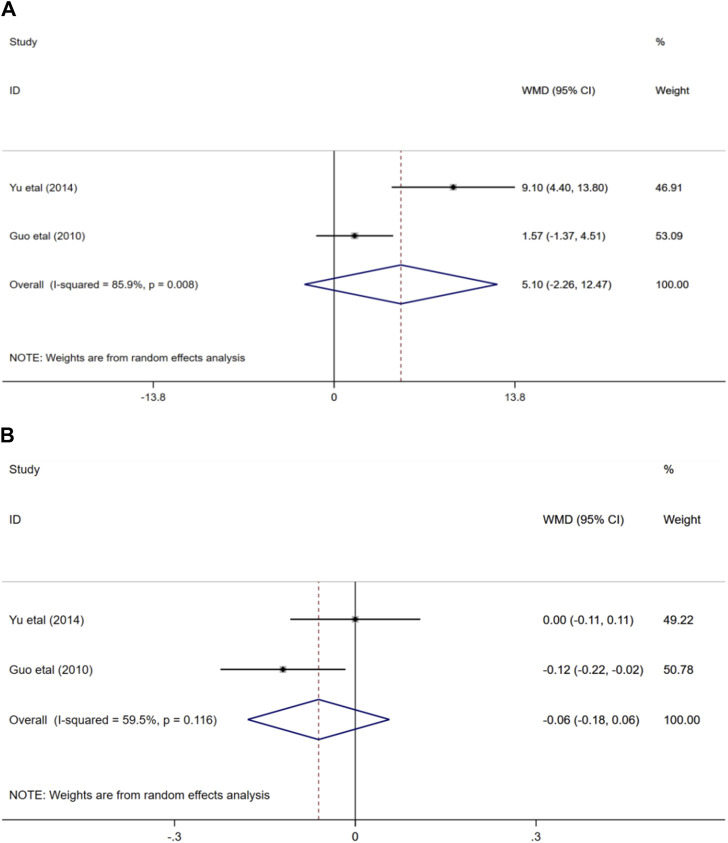
**(A)** Forest plot of mean blood flow velocity of MCA, **(B)** Forest plot of PI of MCA.

Two randomized controlled trials (Guo, 2010; Yu, 2014) evaluated the effects of ginkgo biloba extract combined with donepezil on mean blood flow velocity and pulse index in patients with VaD. In terms of the impact on the mean blood flow velocity of MCA, the result showed that the combination of medication or medication alone could improve the mean blood flow velocity of MCA in participants, and the difference was not statistically significant (WMD = 5.1; 95% CI: −2.26 to 12.47; *p* > 0.05). Similarly, the outcome also demonstrated that there was no statistical difference in the effects of the two treatment options on middle cerebral artery pulse index (WMD = −0.06; 95% CI: −0.18 to 0.06; *p* > 0.05) Figure 9.

#### 3.4.8 Adverse event

**FIGURE 10:**
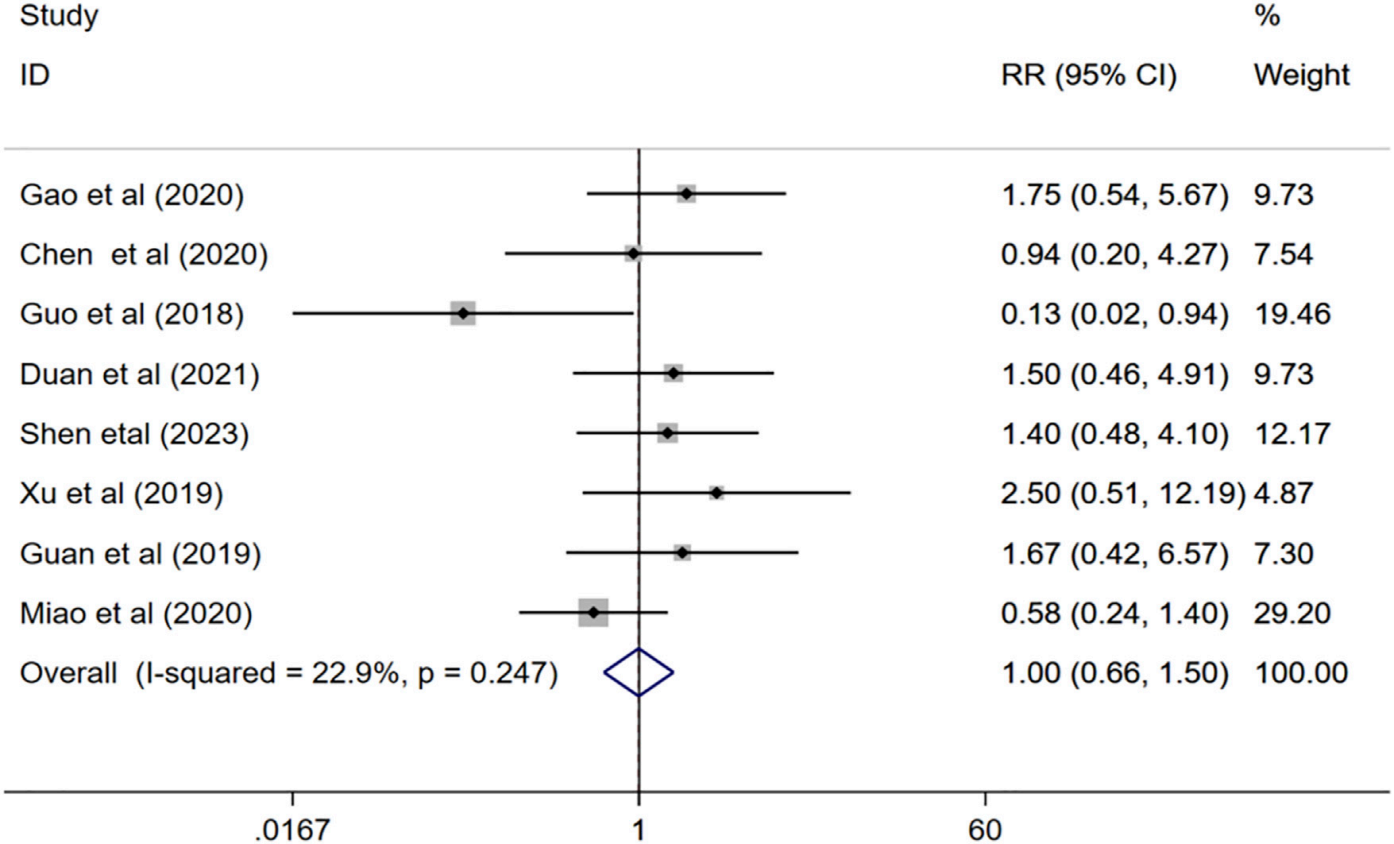
Forest plot of adverse event.

Eight studies (Guo, 2018; Guan, 2019; Xu, 2019; Chen and Li, 2020; Gao et al., 2020; Miao, 2020; Duan et al., 2021; Shen et al., 2023) evaluated the adverse effects of combination therapy for VaD. The meta-analysis of the fix effects model showed that (I^2^ = 22.9%, *p* = 0.247), there is no significant difference between the incidence of adverse event of the experimental group and that of the control group (RR = 1.00, 95%CI 0.66 to 1.50, *p* = 0.981) Figure 10.

### 3.5 Subgroup analysis

To explore the source of heterogeneity, we performed subgroup analysis of total effective rate based on efficacy evaluation criteria, treatment duration and administration route, and subgroup analysis of MMSE score, BI score, and ADL score based on treatment duration and administration route.

The results of subgroup analysis showed that under efficacy evaluation criteria (a 1-point increase in the MMSE score, or a 12% or 15% increase in the MMSE score), treatment duration (4 weeks, 8 weeks or 12 weeks) and administration route (po or ivgtt), the total effective rate of the combination group was significantly higher than that of the donepezil group (Table 2). Regression results further indicated that efficacy evaluation criteria and treatment duration were not the sources of heterogeneity (*p* = 0.606, *p* = 0.237, *p* = 0.158,respectively).

**TABLE 2:** Subgroup analysis of total effective rate based on efficacy evaluation criteria, treatment duration and administration route.

Subgroup	No. of studies	No. of patients	RR (95%CI)	I^2^	P-heterogeneity	*p*-value
**Efficacy evaluation criteria**						
MMSE score increased ≥1	4	386	1.27 (1.15,1.39)	I^2^ = 0.0%	*p* = 0.763	*p* < 0.001
MMSE score increase ≥12%	3	210	1.26 (1.10,1.44)	I^2^ = 66.2%	*p* = 0.052	*p* = 0.001
Clinical symptoms	1	100	1.38 (1.09,1.73)	I^2^ =	P =	*p* = 0.007
MMSE score increase ≥15%	1	158	1.31 (1.09,1.59)	I^2^ =	P =	*p* = 0.004
**Treatment duration**						
4weeks	1	58	1.35 (1.04,1.76)	I^2^ =	P =	*p* = 0.026
8weeks	2	238	1.34 (1.14,1.57)	I^2^ = 0.0%	*p* = 0.754	*p* < 0.001
12weeks	6	558	1.26 (1.16,1.36)	I^2^ = 27.6%	*p* = 0.228	*p* < 0.001
**Administration route**						
po	6	646	1.25 (1.16,1.35)	I2 = 16.1%	*p* = 0.31	*p* < 0.001
ivgtt	3	208	1.41 (1.18,1.69)	I2 = 9.9%	*p* = 0.353	*p* < 0.001

After subgroup analysis of MMSE score based on treatment time, the results showed that MMSE scores were higher in the combination group than in the donepezil group regardless of the duration of treatment at 4, 8, or 12 weeks (*p* = 0.02, *p* < 0.001, *p* < 0.001, respectively) (Table 3). Regression analysis further demonstrated that treatment duration was not the source of heterogeneity (*p* = 0.37). Similarly, subgroup analyses of MMSE scores according to administration route led to higher MMSE scores with combination therapy than with donepezil, whether administered orally or intravenously (*p* < 0.001, *p* < 0.001, respectively) (Table 4).Morever, regression analysis further indicated administration route is not the source of the heterogeneity (*p* = 0.086).

**TABLE 3:** Subgroup analysis of MMSE/BI/ADL scores based on treatment duration.

Subgroup	No. of studies	No. of patients	WMD(95%CI)	I^2^	P-heterogeneity	*p*-value
MMSE score						
4weeks	2	118	3.90 (0.6,7.21)	I^2^ = 90.9	*p* = 0.001	*p* = 0.02
8weeks	2	238	2.43 (1.80,3.06)	I^2^ = 0%	*p* = 0.908	*p* < 0.001
12weeks	9	791	2.87 (2.12,3.62)	I^2^ = 76.3%	*p* < 0.001	*p* < 0.001
BI score						
4weeks	2	126	16.02 (0.55,31.50)	I^2^ = 98.2%	*p* < 0.001	*p* = 0.042
12weeks	4	348	4.82 (0.88,8.75)	I^2^ = 94.5%	*p* < 0.001	*p* = 0.016
ADL score						
8weeks	1	80	7.32 (3.06,11.58)	I^2^ =	P =	*p* = 0.001
12weeks	3	262	10.78 (7.46,14.10)	I^2^ = 84%	*p* = 0.002	*p* < 0.001

**TABLE 4:** Subgroup analysis of MMSE/BI/ADL scores based on administration route.

Subgroup	No. of studies	No. of patients	WMD(95%CI)	I^2^	P-heterogeneity	*p*-value
MMSE score						
po	7	706	2.53 (1.78,3.28)	I2 = 78.3%	*p* < 0.001	*p* < 0.001
ivgtt	6	441	3.61 (2.58,4.64)	I2 = 66.5%	*p* = 0.011	*p* < 0.001
BI score						
po	4	330	4.64 (0.74,8.55)	I2 = 93.3%	*p* < 0.001	*p* = 0.02
ivgtt	2	144	16.09 (0.88,31.3)	I2 = 99.1%	*p* < 0.001	*p* = 0.038
ADL score						
po	1	100	10.11 (7.16,13.07)	I2 =	P =	*p* < 0.001
ivgtt	3	242	8.97 (7.43,10.5)	I2 = 0%	*p* = 0.626	*p* < 0.001

We performed a subgroup analysis of BI scores based on treatment duration, and the results illustrated that after 4 or 12 weeks of treatment, patients in the combination group had higher BI scores than those in the donepezil group (*p* = 0.042, *p* = 0.016, respectively). Moreover, we found in the regression analysis that the BI score increased more after 12 weeks than 4 weeks (*p* = 0.046). In addition, subgroup analysis of BI scores according to the administration route of ginkgo biloba extract showed that BI scores were higher in the combination group than in the donepezil group (*p* = 0.02, *p* = 0.038, respectively) (Table 4). We found in regression analysis that BI scores were higher with intravenous than with oral administration (*p* = 0.044).

However, an analysis of ADL scores based on the same subgroup factors showed that the combination therapy significantly improved ADL scores in patients with VaD at either 8 or 12 weeks of treatment, but the difference was not significant (*p* = 0.378). Besides, based on the administration route of ginkgo biloba extract on ADL scores subgroup analysis, results show that the combination group ADL scores higher than that in donepezil group (*p* < 0.001, *p* < 0.001, respectively). But the interesting thing is, in regression analysis, we found that the ADL score was higher with oral than with intravenous ginkgo biloba extract (*p* < 0.001).

### 3.6 Sensitivity analysis

**FIGURE 11:**
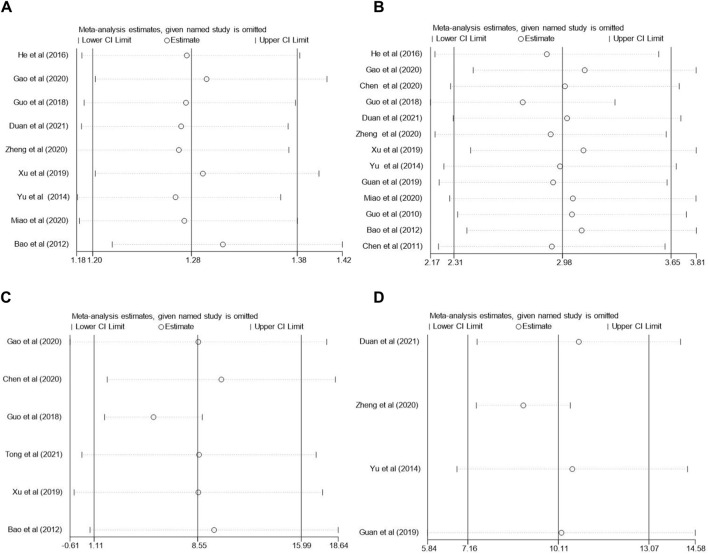
Sensitivity analysis of **(A)** total effective rate, **(B)** MMSE scores, **(C)** BI scores, **(D)** ADL scores.

We performed a sensitivity analysis for the total effective rate, MMSE, BI and ADL scores and assessed the impact of each study on the pooled results through a one-by-one exclusion method. The analysis showed that none of the combined results were significantly affected by any single study. This suggests that the results of this meta-analysis are generally relatively reliable. Sensitivity analysis was shown in following Figure 11.

### 3.7 Publication bias

To ensure the validity of the meta-analysis results, we used funnel plots, Egger’s and Begg’s tests to identify publication bias in total effective rate, MMSE, BI and ADL scores. The results manifested that MMSE, BI and ADL scores had no significant publication bias (*p* > 0.05). On the contrary, there was significant publication bias in the total effective rate (*p* < 0.01). After supplementing four literature with shear compensation method, the conclusions did not change, which further confirmed the reliability of our results (Supplementary Material S2).

## 4 Discussion

Our study found that ginkgo biloba extract combined with donepezil significantly improved the total effective rate, MMSE, BI and ADL scores, and decreased serum homocysteine, plasma viscosity, whole blood high and low tangency viscosity in patients with VaD. The results are similar to previous studies. A meta-analysis of 18 randomized controlled studies (Li et al., 2023) of ginkgo biloba extract combined with donepezil in the treatment of Alzheimer’s disease showed that the combination improved cognitive function in patients, consistent with our findings. A retrospective study concluded that ginkgo biloba extract EGb 761 combined with acetylcholinesterase inhibitors is more effective in the treatment of VaD, which provides real evidence for the treatment of VaD with combined medication (García-Alberca et al., 2022).

Donepezil hydrochloride, it is a kind of acetyl cholinesterase inhibitor, is by far the most commonly used prescription for the treatment of mild-to-moderate AD (42). Existing studies have confirmed that cholinergic deficits are also present in patients with VaD. Therefore, acetylcholinesterase inhibitors may also provide benefit to patients with VaD (Farlow, 2006). Two 6-month randomized, double-blind, placebo-controlled trials investigated the effect of donepezil at either 5 mg/d or 10 mg/d for VaD treatment, each involving more than 600 patients. The results of both studies demonstrated statistically significant dose-related improvements in cognitive function, as measured by changes in ADAS-cog scores, in patients treated with donepezil compared with patients treated with placebo (Wilkinson et al., 2003; Kales et al., 2015). Furthermore, in animal experimental studies, cholinergic dysfunction occurs in mouse models of cerebrovascular injury and in VaD patients (Román, 2005; Román and Kalaria, 2006). A number of studies of cholinergic deficits in cerebral ischemia-associated VaD models have shown persistent reductions in several cholinergic markers. In rats, chronic cerebral hypoperfusion resulting from bilateral common carotid artery occlusion (BCCAO) has been shown to result in the loss of cholinergic neurons, as manifested by reduced choline acetyltransferase (ChAT) and acetylcholinesterase activity (Ni et al., 1995; Tanaka et al., 1996). Another study reported that rats with multiple small emboli have been shown to develop multiple infarcts and show reduced cholinergic markers (Naritomi, 1991). In fact, the application of acetylcholinesterase inhibitors has shown advantages in improving learning and memory impairment and other deficits in VaD patients and VaD animal models (Erkinjuntti et al., 2003; Borlongan et al., 2005; Birks, 2006).

Ginkgo biloba preparations are commonly used in the treatment of ischemic cerebrovascular diseases. A number of studies have shown that ginkgo biloba extract combined with acetylcholinesterase inhibitors is more effective in the treatment of cognitive impairment (Zhang et al., 2019; Zhao et al., 2021). Studies have shown (Zhao et al., 2021) that compared with ginkgo biloba extract (EGb761) or donepezil, the combined application of EGb761 and donepezil has a better anti-amnestic effect on scopolamine-induced cognitive impairment rats, and the mechanism may be that EGb761 enhances the therapeutic effect of donepezil by increasing the pro-cholinergic and antioxidant activities. However, the risks associated with the use of ginkgo biloba preparations are the focus of attention in the medical community. Some researchers have suggested that there is a serious risk of bleeding when ginkgo biloba preparation is combined with anticoagulant or antiplatelet drugs, but existing studies have reported that ginkgo biloba extract EGb761 has no significant effect on the efficacy of aspirin or warfarin combination, and has no adverse effect on safety (Bone, 2008). Due to the limited number of relevant studies and the small sample size and low quality of the available studies, the possibility that ginkgo biloba preparation caused idiosyngenic bleeding events cannot be ruled out based on the available information. In addition, in the existing VaD studies, we found that the serum HCY level was often used as the observation index, and most cross-sectional studies reported that the serum HCY concentration of dementia patients was significantly higher than that of the control group (Clarke et al., 1998; McCaddon et al., 1998; Lehmann et al., 1999). The results of a clinical study showed that HCY concentrations were higher in VaD patients than in controls, suggesting that mildly elevated HCY levels may increase the risk of VaD (McIlroy et al., 2002).

Many studies have used ginkgo biloba preparation combined with conventional western medicine to treat cognitive impairment of cerebral small vessel disease, and the results show that the combined treatment can significantly improve the cognitive function of patients with cerebral small vessel disease and reduce serum HCY, suggesting that ginkgo biloba may treat cognitive impairment of cerebral small vessel disease by reducing HCY (McIlroy et al., 2002; Cao et al., 2018; Hou et al., 2019; Qu, 2019). This is consistent with the results of our study. The mechanism may be related to the regulation of glycogen synthase kinase 3β(GSK-3β) and protein phosphatase 2a (PP2A) activities by ginkgo biloba extract (Zeng et al., 2015).

Subgroup analysis was performed according to efficacy rating criteria and duration of treatment.This study suggested that the evaluation criteria of efficacy did not affect the total effective rate of combination drugs in the treatment of VaD. Besides, treatment duration of 4, 8, 12 weeks or administration route of oral or intravenous did not alter the result of combination therapy on total effective rate and MMSE score. Among them, we found that the treatment duration of 12 weeks was more beneficial to the increase of BI score in patients with VaD than that of 4 weeks, whereas there was little difference in improvement in ADL scores between 12 and 8 weeks of treatment. More interestingly, the results showed that the BI score was higher when ginkgo biloba extract was administered intravenously than when it was administered orally, but the ADL score was higher when it was administered orally than when it was administered intravenously. Therefore, in the treatment of vascular dementia, attention should be paid to shorten the treatment time, reduce the treatment cost, and rationally allocate medical resources. However, due to the limited number of included references, the results should be interpreted with caution.

The limitations of this study are as follows: First, the articles included in this study were all single-center studies and carried out in China, which may bring resistance to the international promotion of combination drugs; Second, the heterogeneity is high, which may be related to the differences of drug dosage form (tablets, capsules, injections), active ingredient content (terpenolides: ginkgolides, ginkgolactones; ginkgo flavonoids), medication route (oral, intravenous drip), and age, gender and race of the study population.

## 5 Conclusion

In summary, the available evidence suggests that compared with Donepezil alone, ginkgo biloba extract combined with Donepezil is more effective in the treatment of vascular dementia and has a better effect on cognitive function and daily living ability in these patients. In addition, combination therapy may decrease hemorheological indicators and serum HCY content in patients with vascular dementia, but no combination therapy was found to directly improve cerebral hemodynamic parameters in participants. Multicenter randomized controlled studies with high quality and large samples are needed for validation.

## Data Availability

The original contributions presented in the study are included in the article/Supplementary Material, further inquiries can be directed to the corresponding author.
