# Supplementary material for: Efficacy and safety of ginkgo biloba extract combined with donepezil hydrochloride in the treatment of Chinese patients with vascular dementia: A systematic review meta-analysis
Source: Front Pharmacol. 2024 Jul 3;15:1374482. doi: 10.3389/fphar.2024.1374482 (PMC11251972; doi:10.3389/fphar.2024.1374482)
Supplement: Supplementary file 1 [file DataSheet2.docx]

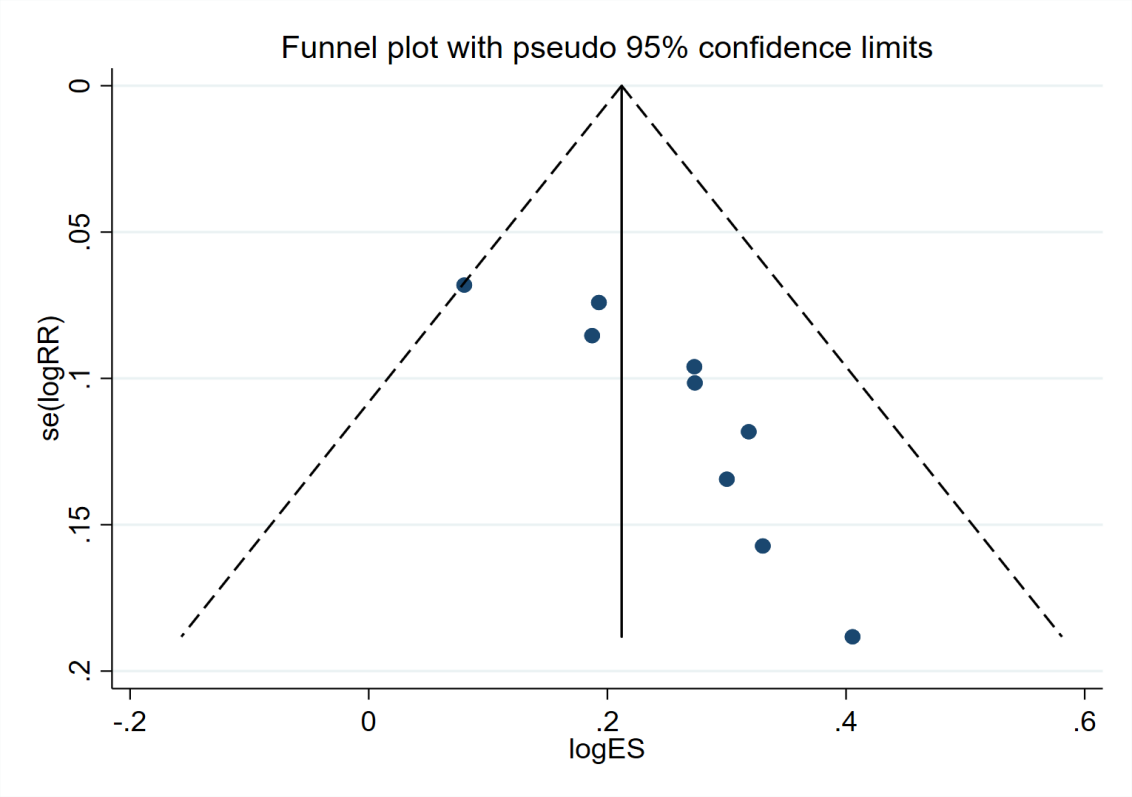

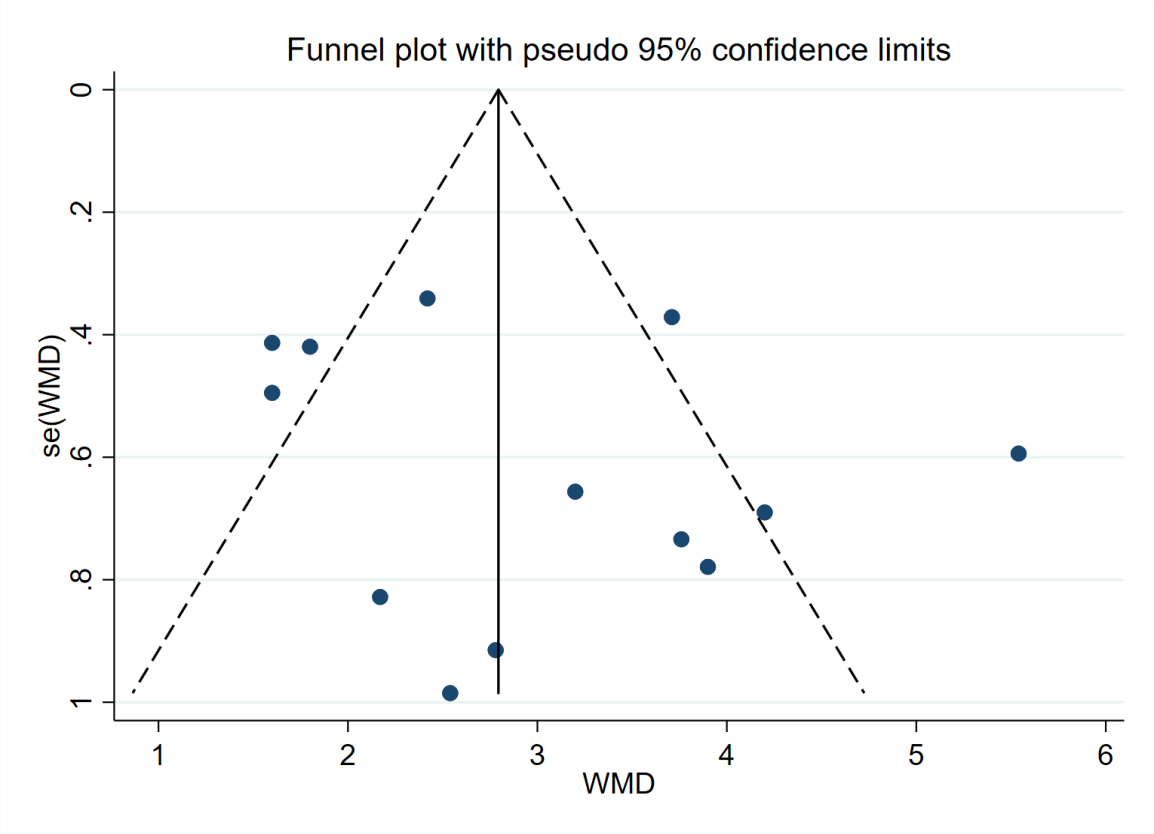


A: Funnel plot of total effect rate. B: Funnel plot of MMSE scroes.


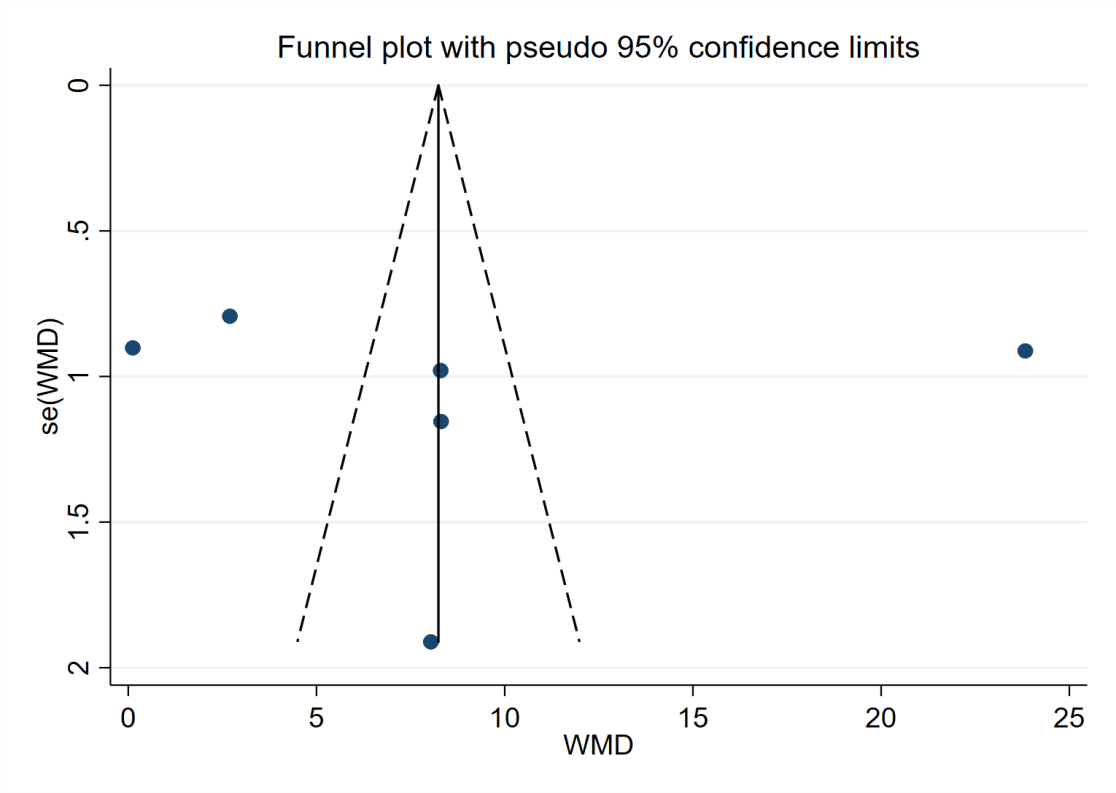

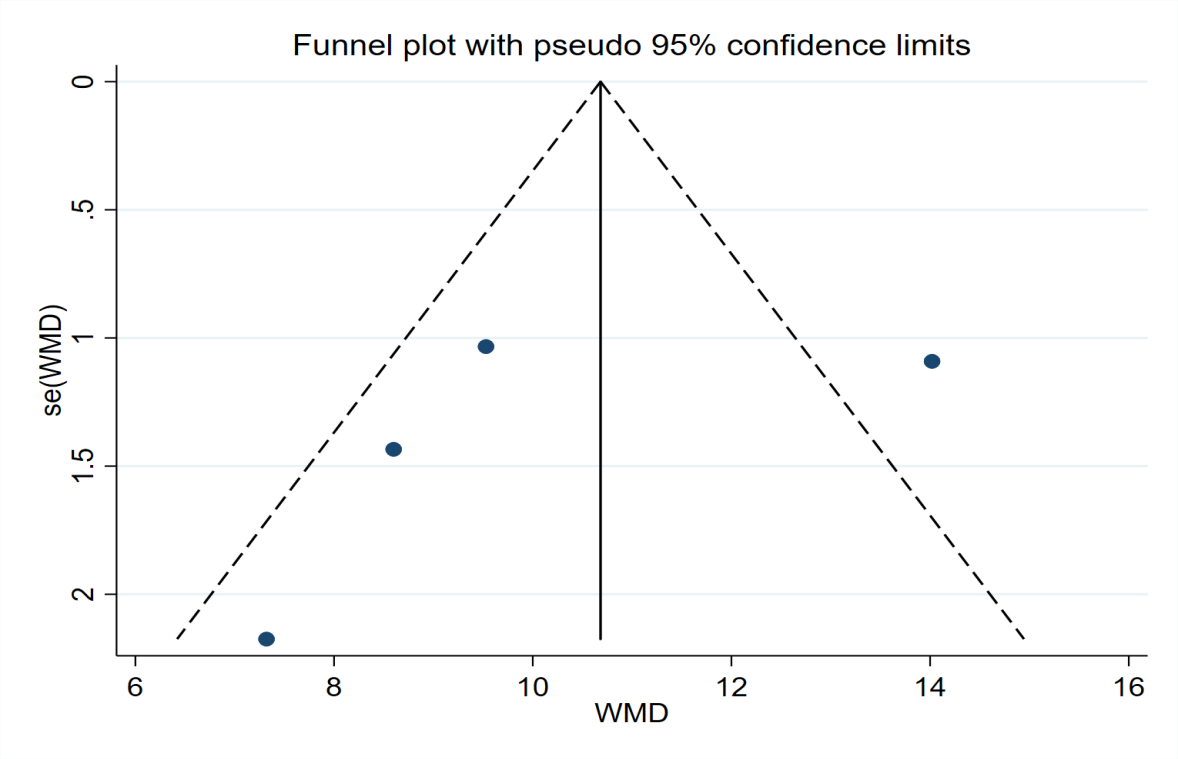


C: Funnel plot of BI scroes. D: Funnel plot of ADL scroes.
