# Supplementary material for: Efficacy and safety of ginkgo biloba extract combined with donepezil hydrochloride in the treatment of Chinese patients with vascular dementia: A systematic review meta-analysis
Source: Front Pharmacol. 2024 Jul 3;15:1374482. doi: 10.3389/fphar.2024.1374482 (PMC11251972; doi:10.3389/fphar.2024.1374482)
Supplement: Supplementary file 2 [file DataSheet1.docx]

# Supplementary Material 1: Literature search strategy

**1.Pubmed**

| Search number | Query |
| --- | --- |
| #1 | "Dementia, Vascular"[Mesh] |
| #2 | ((((((((((Vascular Dementias[Title/Abstract]) OR (cerebrovascular[Title/Abstract])) OR (post-stroke dementia[Title/Abstract])) OR (post-stroke cognitive impairment[Title/Abstract])) OR (vascular cognitive impairment[Title/Abstract])) OR (Binswanger*[Title/Abstract])) OR (CADASIL[Title/Abstract])) OR (Dement*[Title/Abstract])) OR (VaD[Title/Abstract])) OR (VD[Title/Abstract])) OR (VCI[Title/Abstract]) |
| #3 | ("Dementia, Vascular"[Mesh]) OR (((((((((((Vascular Dementias[Title/Abstract]) OR (cerebrovascular[Title/Abstract])) OR (post-stroke dementia[Title/Abstract])) OR (post-stroke cognitive impairment[Title/Abstract])) OR (vascular cognitive impairment[Title/Abstract])) OR (Binswanger*[Title/Abstract])) OR (CADASIL[Title/Abstract])) OR (Dement*[Title/Abstract])) OR (VaD[Title/Abstract])) OR (VD[Title/Abstract])) OR (VCI[Title/Abstract])) |
| #4 | "Donepezil"[Mesh] |
| #5 | ((((Eranz[Title/Abstract]) OR (E2020[Title/Abstract])) OR (E-2020[Title/Abstract])) OR (Donepez*[Title/Abstract])) OR (Aricept[Title/Abstract]) |
| #6 | ("Donepezil"[Mesh]) OR (((((Eranz[Title/Abstract]) OR (E2020[Title/Abstract])) OR (E-2020[Title/Abstract])) OR (Donepez*[Title/Abstract])) OR (Aricept[Title/Abstract])) |
| #7 | "Ginkgo biloba"[Mesh] |
| #8 | (((((ginkgo biloba*[Title/Abstract]) OR (ginkgo leaf extract[Title/Abstract])) OR (ginkgo*[Title/Abstract])) OR (ginko*[Title/Abstract])) OR (Maidenhair[Title/Abstract])) OR (yinxing*[Title/Abstract]) |
| #9 | ("Ginkgo biloba"[Mesh]) OR ((((((ginkgo biloba*[Title/Abstract]) OR (ginkgo leaf extract[Title/Abstract])) OR (ginkgo*[Title/Abstract])) OR (ginko*[Title/Abstract])) OR (Maidenhair[Title/Abstract])) OR (yinxing*[Title/Abstract])) |
| #10 | ((("Dementia, Vascular"[Mesh]) OR (((((((((((Vascular Dementias[Title/Abstract]) OR (cerebrovascular[Title/Abstract])) OR (post-stroke dementia[Title/Abstract])) OR (post-stroke cognitive impairment[Title/Abstract])) OR (vascular cognitive impairment[Title/Abstract])) OR (Binswanger*[Title/Abstract])) OR (CADASIL[Title/Abstract])) OR (Dement*[Title/Abstract])) OR (VaD[Title/Abstract])) OR (VD[Title/Abstract])) OR (VCI[Title/Abstract]))) AND (("Donepezil"[Mesh]) OR (((((Eranz[Title/Abstract]) OR (E2020[Title/Abstract])) OR (E-2020[Title/Abstract])) OR (Donepez*[Title/Abstract])) OR (Aricept[Title/Abstract])))) AND (("Ginkgo biloba"[Mesh]) OR ((((((ginkgo biloba*[Title/Abstract]) OR (ginkgo leaf extract[Title/Abstract])) OR (ginkgo*[Title/Abstract])) OR (ginko*[Title/Abstract])) OR (Maidenhair[Title/Abstract])) OR (yinxing*[Title/Abstract]))) |

**2.Cochrane**

| Search number | Query |
| --- | --- |
| #1 | MeSH descriptor: [Dementia, Vascular] explode all trees |
| #2 | (Vascular Dementias):ti,ab,kw OR (cerebrovascular):ti,ab,kw OR (post-stroke dementia):ti,ab,kw OR (post-stroke cognitive impairment):ti,ab,kw OR (vascular cognitive impairment):ti,ab,kw |
| #3 | #3(Vascular Dementia):ti,ab,kw OR (Binswanger*):ti,ab,kw OR (CADASIL):ti,ab,kw OR (Dement*):ti,ab,kw OR (VaD):ti,ab,kw |
| #4 | (VD):ti,ab,kw OR (VCI):ti,ab,kw |
| #5 | #1 or #2 or #3 or #4 |
| #6 | MeSH descriptor: [Donepezil] explode all trees |
| #7 | (donepezil):ti,ab,kw OR (Eranz):ti,ab,kw OR (E2020):ti,ab,kw OR (E-2020):ti,ab,kw OR (Donepez*):ti,ab,kw |
| #8 | (Aricept):ti,ab,kw |
| #9 | #7 or #8 |
| #10 | MeSH descriptor: [Ginkgo biloba] explode all trees |
| #11 | (ginkgo biloba):ti,ab,kw OR (ginkgo biloba*):ti,ab,kw OR (ginkgo leaf extract):ti,ab,kw OR (ginkgo*):ti,ab,kw OR (ginko*):ti,ab,kw |
| #12 | (Maidenhair):ti,ab,kw OR (yinxing*):ti,ab,kw |
| #13 | #11 or #12 |
| #14 | #5 and #9 and #13 |

**3.Embase**

| Search number | Query |
| --- | --- |
| #1 | 'multiinfarct dementia'/exp |
| #2 | 'vascular dementias':ab,ti OR 'vascular dementia':ab,ti OR cerebrovascular:ab,ti OR 'post-stroke dementia':ab,ti OR 'post-stroke cognitive impairment':ab,ti OR 'vascular cognitive impairment':ab,ti OR binswanger*:ab,ti OR cadasil:ab,ti OR dement*:ab,ti OR vad:ab,ti OR vd:ab,ti OR vci:ab,ti |
| #3 | 'donepezil'/exp |
| #4 | eranz:ab,ti OR e2020:ab,ti OR 'e 2020':ab,ti OR donepez*:ab,ti OR aricept:ab,ti |
| #5 | 'ginkgo biloba'/exp |
| #6 | 'ginkgo biloba*':ab,ti OR 'ginkgo leaf extract':ab,ti OR ginkgo*:ab,ti OR ginko*:ab,ti OR maidenhair:ab,ti OR yinxing*:ab,ti |
| #7 | #1 OR #2 |
| #8 | #3 OR #4 |
| #9 | #5 OR #6 |
| #10 | #7 AND #8 AND #9 |

**4.Web of science**

| Search number | Query |
| --- | --- |
| #1 | Vascular Dementia (Topic) OR Vascular Dementias (Topic) OR Vascular Dementia (Topic) OR cerebrovascular (Topic) OR post-stroke dementia (Topic) OR post-stroke cognitive impairment (Topic) OR vascular cognitive impairment (Topic) OR Binswanger* (Topic) OR CADASIL (Topic) OR Dement* (Topic) OR VaD (Topic) OR VD (Topic) OR VCI (Topic) |
| #2 | donepezil (Topic) OR Eranz (Topic) OR E2020 (Topic) OR E-2020 (Topic) OR Donepez* (Topic) OR Aricept (Topic) |
| #3 | Ginkgo biloba (Topic) OR ginkgo biloba* (Topic) OR ginkgo leaf extract (Topic) OR ginkgo* (Topic) OR ginko* (Topic) OR Maidenhair (Topic) OR yinxing* (Topic) |
| #4 | #1 AND #2 AND #3 |

Four Chinese databases[the China National Knowledge Infrastructure (CNKI), Wanfang DATA, the Chongqing VIP Database (VIP), China Biomedical Database (CBM,Sinomed)] were manually searched，and here is an example of a Sinomed search:

**5.Sinomed**

| Search number | Query |
| --- | --- |
| #1 | 血管性痴呆 |
| #2 | ( "血管性痴呆"[常用字段:智能] OR "血管性认知障碍"[常用字段:智能] OR "VaD"[常用字段:智能] OR "VD"[常用字段:智能]) |
| #3 | (#2) OR (#1) |
| #4 | "多奈哌齐"[不加权:扩展] |
| #5 | ( "多奈哌齐"[常用字段:智能] OR "安理申"[常用字段:智能] OR "胆碱酯酶抑制剂"[常用字段:智能]) |
| #6 | (#5) OR (#4) |
| #7 | ("银杏叶"[不加权:扩展])OR "银杏叶口服液"[不加权:扩展] OR "银杏叶"[不加权:扩展] |
| #8 | ( "银杏叶"[常用字段:智能] OR "银杏"[常用字段:智能] OR "银杏叶口服液"[常用字段:智能] OR "银杏叶片提取物"[常用字段:智能]) |
| #9 | (#8) OR (#7) |
| #10 | (#9) AND (#6) |
